# Capnodynamic assessment of mixed venous oxygen saturation in a porcine experimental endotoxemic model

**DOI:** 10.1038/s41598-024-77483-7

**Published:** 2024-11-05

**Authors:** Anders Svedmyr, Joakim Hedov, Miklos Lipcsey, Mats Wallin, Magnus Hallbäck, Per-Arne Lönnqvist, Jacob Karlsson

**Affiliations:** 1 Department of Physiology and Pharmacology, (FYFA), Karolinska Institute, C3, PA Lönnqvist Group- Section of Anesthesiology and Intensive Care, Anestesi-och Intensivvårdsavdelningen,, 171 76 Stockholm, Sweden; 2 Pediatric Perioperative Medicine and Intensive Care, Karolinska University Hospital, Eugenivägen 23, 171 64 Stockholm, Sweden; 3 Maquet Critical Care AB, Röntgenvägen 2, 171 06 Solna, Sweden; 4 Department of Surgical Sciences, Section of Anesthesiology and Intensive Care, Uppsala University, Uppsala, Sweden; 5 Hedenstierna Laboratory, Department of Surgical Sciences, Uppsala University, Uppsala, Sweden

**Keywords:** SvO_2_, Mixed venous saturation, Sepsis, Septic shock, Hemodynamic monitoring, Non-invasive, Circulation, Blood flow, Experimental models of disease, Sepsis

## Abstract

Sepsis continues to be a major cause of death and illness globally, posing significant challenges for healthcare professionals. In the pursuit of more accurate and timely monitoring tools, the concept of capnodynamically derived mixed venous oxygen saturation (Capno-SvO_2_) has emerged as a promising method. Capno-SvO_2_ provides a non-invasive way to assess and track SvO_2_ and could serve as an additional tool alongside more invasive methods like the pulmonary artery catheter. This could potentially be of great value in the care of critically ill patients with sepsis, where alternative minimal invasive monitoring methods may vary in reliability. The aim of the current study was to compare capno-SvO_2_ against values obtained through pulmonary artery blood sample CO-oximetry and continuous fiberoptic SvO_2_ monitoring, using a well-established porcine experimental sepsis model. Anesthetized pigs were exposed to a standardized endotoxin infusion sepsis protocol, followed by a series of maneuvers typically applied in sepsis care. Simultaneous recordings were done throughout the experiment for all three monitoring methods. Bland–Altman analysis corrected for repeated measurements was used to assess the agreement of absolute values between the paired recording of CO-oximetry and Capno-SvO_2_ as well as between CO-oximetry and fiberoptic SvO_2_. The ability of Capno-SvO_2_ and fiberoptic SvO2 to track changes was assessed by concordance rate. A total of 10 animals and 275 paired datapoints were included in the study. The majority of the animals displayed pronounced hemodynamical instability in response to endotoxin exposure and subsequent treatment interventions. Analysis of all paired data points showed a bias between Capno-SvO_2_ and CO-oximetry SvO_2_ of + 1% with 95% limits of agreement of -14% to + 17%. The corresponding numbers for fiberoptic SvO_2_ and CO-oximetry SvO_2_ were -4% and -15% to + 8%. The concordance rate as compared to CO-oximetry, were 97% and 93% for Capno-SvO_2_ and fiberoptic SvO_2_, respectively. In this experimental sepsis model, continuous, non-invasive Capno-SvO_2_ generates average absolute values comparable to the gold standard CO-oximetry albeit with relatively wide limits of agreement. Capno-SvO_2_ displayed a concordance rate of 97% against CO-oximetry and exhibits better trending ability compared to invasive fiberoptic SvO_2_.

## Introduction

Sepsis remains a leading cause of mortality and morbidity worldwide, challenging clinicians with its complex pathophysiology and dynamic clinical course. In the pursuit of more accurate and timely monitoring tools, the concept of capnodynamically derived mixed venous oxygen saturation (capno-SvO_2_) has emerged as a promising method^1–3^. Capno-SvO_2_ offers a non-invasive mean of monitoring mixed venous oxygen saturation and may be used as a supplement to more invasive SvO_2_ methods such as Pulmonary Artery Catheter (PAC).

Ever since the introduction of the pulmonary artery catheter in 1970 by Swan and Ganz, mixed venous oxygen saturation (SvO_2_) has been used as a marker of balance between oxygen delivery and consumption in critically ill patients^4^. Even though previous literature indicates a co-variability between low SvO_2_ and poorer outcome, most large, controlled trials have shown variable benefits from the use of PAC regarding clinical outcomes, such as mortality or ICU length of stay^5–8^. In addition, concerns regarding catheter-related risks and adverse events still exist^9^. Recent studies have however again pointed towards a potential beneficial role for PAC in advanced hemodynamic monitoring, provided that the benefits outweigh the risks^10^. Against this background, it would be valuable to investigate alternative non-invasive techniques intended to monitor mixed venous oxygen saturation.

While previous studies of Capno-SvO_2_ have been promising in various experimental and clinical settings of both minor and major hemodynamic challenges, the validation against gold standard CO-oximetry in a sepsis model, is lacking. Sepsis is characterized by a state of disturbed mitochondrial function, oxygen extraction and carbon dioxide removal which all together challenges the basis of the capnodynamic method^2^.

Thus, the primary objective of this study was to compare absolute values of capno-SvO_2_ against values obtained through pulmonary artery blood sample CO-oximetry and continuous fiberoptic SvO_2_ monitoring, using a well-established porcine experimental endotoxemic model^2^. In addition, capno-SvO_2_ was validated for its ability to detect a change in mixed venous oxygen saturation in response to the development of sepsis and subsequent typical therapeutic interventions.

## Methods

This porcine study was carried out at the Hedenstierna Laboratory, Uppsala University, Uppsala, Sweden, in 2023. Authorization was granted by Uppsala Animal Ethics Committee (Uppsala, Sweden case number 5.2.18–8927/16, chairperson Erik Göransson on August 27, 2021). The animals were handled in accordance with the animal experimentation guidelines of the Uppsala Animal Ethics Committee and Animal Research: Reporting of In Vivo Experiments (ARRIVE) guidelines.

The animals were kept in a light- and temperature-controlled environment, with unlimited access to tap water and food on a standardized schedule. The experiments were done during daytime hours.

### Anesthesia protocol

The pigs were anaesthetized as previously described^11^ and mechanically ventilated in a volume-controlled mode (Servo-I; Maquet, Solna, Sweden), with target tidal volume of 10 ml kg^-1^ and fraction of inspired oxygen (FiO_2_) 0.3. Positive end-expiratory pressure (PEEP) was kept at 5 cm H_2_O after an initial 2-min period of lung expansion using PEEP 10 cm H_2_O and constant driving pressure as previously described. After the lung expansion manoeuvre, an air test using FiO_2_ 0.21 was performed and repeated after an additional lung expansion manoeuvre if necessary, aiming for sustained pulse oximetry saturation > 97% as indicative of open lung conditions^12^. The animals were given a bolus of Ringers’ acetate solution 20 ml kg^-1^ after anaesthesia induction and thereafter kept on maintenance infusion of glucose 25 mg ml ^-1^ 8 ml kg^-1^ h^-1^ and Ringer’s acetate solution 10 ml kg^-1^ h^-1^. Exhaled CO_2_ was measured by a mainstream infrared CO_2_ sensor (Capnostat-3; Respironics Inc, Wallingford, CT), and ventilation airflow was registered through the regular flow sensor of the servo-I ventilator. Volumetric capnography was utilised for calculating the CO_2_ elimination rate (VCO_2_), as described in previous studies^13^.

Adequate anesthetic depth and analgesic level were tested regularly during the experiment according to standard procedures of the laboratory as previously described^14^. The animals were fitted with standard laboratory monitoring devices described in detail in previous studies^11^. In addition a 7,5F pulmonary artery catheter with fibreoptic SvO_2_ monitoring (Swan-Ganz pulmonary artery catheter, model 774F75; Edwards Lifesciences, Irvine CA; USA) and a 5F femoral artery cannula for transpulmonary thermodilution cardiac output monitoring (PICCO2™, Pulsion Medical Systems, Munich, Germany), were also included as well as an additional 7,5F pulmonary artery introducer sheath for rapid fluid administration.

The animals were given a bolus dose of intravenous heparin 5000 U (LEO pharma) to minimize the risk of clotting due to the extensive intravascular monitoring setup.

Blood gases were analyzed via blood samples using a CO-oximeter calibrated specifically for porcine hemoglobin (ABL800 Radiometer AS, Copenhagen, Denmark). This also provided the hemoglobin level, necessary for the capnodynamic calculations as well as for calibrations of fiberoptic SvO_2_.

### Assessment of Capno-SvO2

The continuous Capno-SvO_2_ method, as detailed in previous studies^1,2^, is based on the differential Fick’s principle. This approach combines the continuous estimation of Effective Pulmonary Blood Flow (EPBF) and oxygen consumption (VO_2_) within a modified Fick’s equation as illustrated in Eq. 1–3 below^2^. EPBF is estimated by using a specialized breathing pattern involving variations in respiratory rate. A software (developed for research purposes) in the ventilator produces six breaths with normal rate, followed by three breaths with an approximately 2–3 s expiratory pause. This ventilation pattern induces minor fluctuations in alveolar CO_2_ concentration and elimination, related to the pulmonary blood flow participating in gas exchange (i.e., EPBF). This enables a breath-by-breath estimation of EPBF^15–17^. (Figures describing the breathing pattern and the setup are available as supplementary material).

The other main determinant of Capno-SvO_2_, VO_2_, can be calculated by continuous volumetric capnography measurement of VCO_2_ combined with the Respiratory Quotient as described in Eq. 1 below.

1 $$V{O}_{2}=\frac{VC{O}_{2}}{RQ}$$

(Eq. 1, where VO_2_ is oxygen consumption in ml min^-1^, VCO_2_ is CO_2_ production rate in ml min^-1^, and RQ is the Respiratory Quotient).

The VCO_2_ in Eq. 1 is computed as a moving mean value of breath-by-breath CO_2_ elimination measured over a 20-min period to achieve stable VCO_2_ values, reflecting an assumed stable metabolism.

Utilizing the classic Fick’s equation^18^, the oxygen content in mixed venous blood (CvO_2_) can be derived:

2 $$C{O}_{EPBF}=\frac{V{O}_{2}}{Cc{O}_{2}-Cv{O}_{2}}$$

Equation 2, where CO_EPBF_ is Effective Pulmonary Blood Flow in L min^-1^, VO_2_ is oxygen consumption in ml min^-1^, CcO_2_ is pulmonary end capillary oxygen content in ml L^-1^, and CvO_2_ is pulmonary mixed venous oxygen content in ml L^-1^. The pulmonary end capillary blood is assumed to be saturated and in equilibrium with the partial pressure of oxygen in alveolar gas, which was calculated from the alveolar gas equation.

Equation 2 can subsequently be rearranged to obtain CvO_2_.

3 $$Cv{O}_{2}=Cc{O}_{2}-\frac{V{O}_{2}}{C{O}_{EPBF}}$$

CvO_2_ from Eq. 3 is then used to calculate SvO_2_, employing the solubility constant for oxygen in blood plasma, Hüfner’s constant, and the hemoglobin value, as described and validated in previous publications, thereby generating breath-by-breath estimations of SvO_2_^1,2^.

### Respiratory quotient

For this study, we used the RQ 0.97 calculated in a previous porcine experimental study using a similar setup and cohort of animals^1^.

### Study protocol

Simultaneous recordings of all three modalities for SvO_2_ i.e. capno-SvO_2_, CO-oximetry SvO_2_ and fiberoptic SvO_2_ were done throughout the protocol.

After the initial preparation, the animals were allowed a 20-min stabilization period before the protocol was started. A blood sample was drawn from the pulmonary artery and the fiberoptic SvO_2_ monitoring system was calibrated according to the manufacturer’s description. Then a series of five consecutive readings of all three SvO_2_ methods was done, five minutes apart, to calculate their respective inherent precision. After that, just prior to the initiation of the sepsis protocol, a baseline sample was taken. After the precision measurements, the protocol consisted of two sections, outlined below, where all three SvO_2_ methods (i.e., CO-oximetry, fiberoptic and capnodynamics) were recorded in parallel.

1. SvO_2_ reaction under the development of sepsis.
2. The effects on SvO_2_ of standardized treatment actions in sepsis.

### Experimental endotoxemic model

A well-established experimental endotoxemic protocol was used based on an intravenous challenge of endotoxin (lipo-polysaccharide, LPS), given at a rate of 2 µg × kg^−1^ × h^−1^ throughout the rest of the experiment. The LPS infusion causes a hemodynamically unstable situation, intended to mimic the accelerating development of sepsis^19^. A standardized goal-directed intervention protocol was used during this phase of the experiment, to ensure the survival of the subjects for the second part of the protocol (see appendix 1 for goal directed protocol)^20^.

For the first hour after initiation of the LPS infusion, SvO_2_ data was recorded every 10 min to capture the development of sepsis and its effect on mixed venous oxygen saturation.

After this initial hour, the animals were allowed approximately one hour of stabilization before the second part of the protocol was carried out. Before commencing the second part of the study protocol, we strived to reach the baseline hemodynamic status recorded prior to the start of LPS challenge since some of the ongoing interventions associated with the goal-directed stabilization (i.e., noradrenaline-infusion and high inspired fraction of oxygen) could interfere with the second part of the study protocol. After a recalibration of the fiberoptic module, the second part of the protocol was started, intended to simulate typical interventions associated with sepsis stabilization in the intensive care setting. Six different challenges were carried out in the order described below.

1. **Crystalloid bolus**: 15 ml kg^-1^ of lactated Ringer solution was administered through the PAC introducer over 3–5 min. Simultaneous recordings of all three SvO_2_ monitoring modalities were done just prior to the infusion and twenty minutes after termination of the fluid bolus.
2. **Stepwise increase and decrease in FiO**_**2**_: The oxygen level was changed every fifth minute from 0.3 – 0.5 – 0.8 – 1.0 –0.3. Recordings of SvO_2_ were made just before each change in FiO_2_.
3. **Alteration of positive end-expiratory pressure (PEEP**): The last recording from the previous step (i.e., 5 cm H_2_O in step 2 FiO_2_ 0.3) was used as baseline. PEEP was then altered from 10 – 15 – 5 cmH_2_O and SvO_2_ recordings were done at the end of each step after a short stabilization period (approximately 1–2 min)
4. **Dobutamine**: The last reading from the previous step was used for baseline. A central dobutamine infusion running at 15 mcg kg^-1^ min^-1^ was started and new recordings were made after 15 min after which the infusion was terminated.
5. **Norepinephrine**: A new baseline was recorded 15 min after termination of dobutamine (to allow wash out of dobutamine and return to baseline hemodynamics) and a central norepinephrine infusion of 0.1 mcg kg^-1^ min^-1^ was started. If norepinephrine was already running due to hemodynamic instability (as per the goal-directed treatment plan described in appendix 1), the infusion rate was increased by 0.1 mcg kg^-1^ min^-1^. A recording was done after 10 min, and the infusion was then stopped or returned to the original infusion rate used prior to the increase
6. **Hemorrhage**: A new baseline was done 10 min after termination of norepinephrine infusion. 15 ml kg^-1^ blood was then withdrawn from the PA introducer sheath and stored in a plastic bag intended for donor blood, containing 5000 U of Heparin (LEO Pharma). SvO_2_ values were recorded 10 min after completion of the hemorrhage.
7. **Restitution of whole blood**: the blood was transfused back over a 5–10 min period and SvO_2_ data were recorded 10 min after completed transfusion.

After the experiment, the animals were euthanized using intravenous potassium chloride (KCl: 100 mmol) via the central venous catheter as per established standards at the laboratory.

### Statistical analysis

Raw data for absolute values of all three SvO_2_ methods (CO-oximetry, Capno-SvO_2_ and fibreoptic SvO_2_), as well as paired differences between Capno-SvO_2_ and CO-oximetry and between fibreoptic SvO_2_ and CO-oximetry, were controlled for normal distribution by D’Agostino and Pearson test and visual inspection of the corresponding histograms. Values are presented as mean and 95% confidence interval (CI).

The first five measurements, during hemodynamically stable baseline conditions, were used for the calculation of inherent precision for each SvO_2_ method respectively. Inherent precision was defined as 2 times the coefficient of variation (CV = SD/mean)^21^ and was in turn used to quantify the least significant change (LSC) for the reference method CO-oximetry. The LSC reflects the minimum change measured and recognized as a true change^21^. LSC for the reference method was then used to determine the exclusion zone in the concordance analysis as previously described^2^.

Bland–Altman analysis corrected for repeated measurements was used to assess the agreement of absolute values between the paired recording of CO-oximetry and Capno-SvO_2_ as well as between CO-oximetry and fibreoptic SvO_2_. Bias was defined as the mean difference between the tested methods and the reference method^22,23^. Limits of agreement were used when presenting the spread of included data points and the corresponding 95% CI calculated. No sample size calculation was done, rather a sample size of 10 animals was used based on previous studies with a similar setup. 12 animals were included for upward margin^1^. Acceptable limits of agreement against CO-oximetry were set to ± 15% based on previous publications on the performance of fiberoptic SvO_2_ monitoring which represents an alternative to Capno-SvO_2_ monitoring^24–26^.

The ability of Capno-SvO_2_ and fibreoptic SvO_2_ to track changes in SvO_2_ compared to the reference method, was assessed by calculating the concordance rate, i.e. the percentage of data points moving in the same direction when comparing two different techniques^27^. Datapoints representing the anticipated extremes of SvO_2_ within each intervention were identified, i.e., FiO_2_ 0.3 and 1.0, or the datapoints just before and after whole blood transfusion. Recorded changes between these extremes, before and after each hemodynamic intervention, are displayed in a 4-quadrant plot and used for the calculation of the concordance rate. The concordance rate is presented with a corresponding 95% CI as previously described^28^. A concordance rate of > 92% was considered clinically useful^27^.

GraphPad Prism (version 9.0.0 for Windows, GraphPad Software, San Diego, CA, USA) and Medcalc Statistical Software version 16.8.4 (MedCalc Software, Ostend, Belgium) was used for statistical calculations and Microsoft Excel for Mac 2020 version 16.41 for data handling.

## Results

Twelve domestic-breed pigs of both sexes (median weight 31.5 kg, range 28.7–33.3 kg, 6–8 weeks of age), from the same breeding colony were used. Two of the animals (on two consecutive days) were highly hemodynamically unstable. One of these two animals died early during the experiment and the second animal suffered a sustained severe pulmonary hypertensive reaction throughout the study. They were both excluded from the analyses since the validity of the data collected was deemed compromised by the ongoing resuscitation of the animals and by the lack of hemodynamic stability before the start of the protocol. Both animals were investigated on a separate week, not related to the remaining subjects. The remaining 10 animals all survived the experiment, and their data were included in the study. Under step 2 in the second part of the protocol, the FiO_2_ 1.0 step was introduced starting from the sixth animal to enable the calculation of any pulmonary shunt (data not shown) and therefore contains data from five animals in total.

### Calculated inherent precision and least significant change

The inherent precision was found to be ± 11% for Co-oximetry SvO_2_ and ± 6% and ± 7% for Capno-SvO_2_ and fiberoptic SvO_2_, respectively, which is in line with previous publications^1,2^. The LSC was calculated to 16%, corresponding to a minimal change in SvO_2_ of 10 percentage points, i.e., a change in the reference method CO-oximetry SvO_2_ has to be more than 10 percentage points in magnitude in order to be regarded as a real change and not attributed to imprecision in measurement^21^.

### Response to hemodynamic challenges

**Fig. 1:**
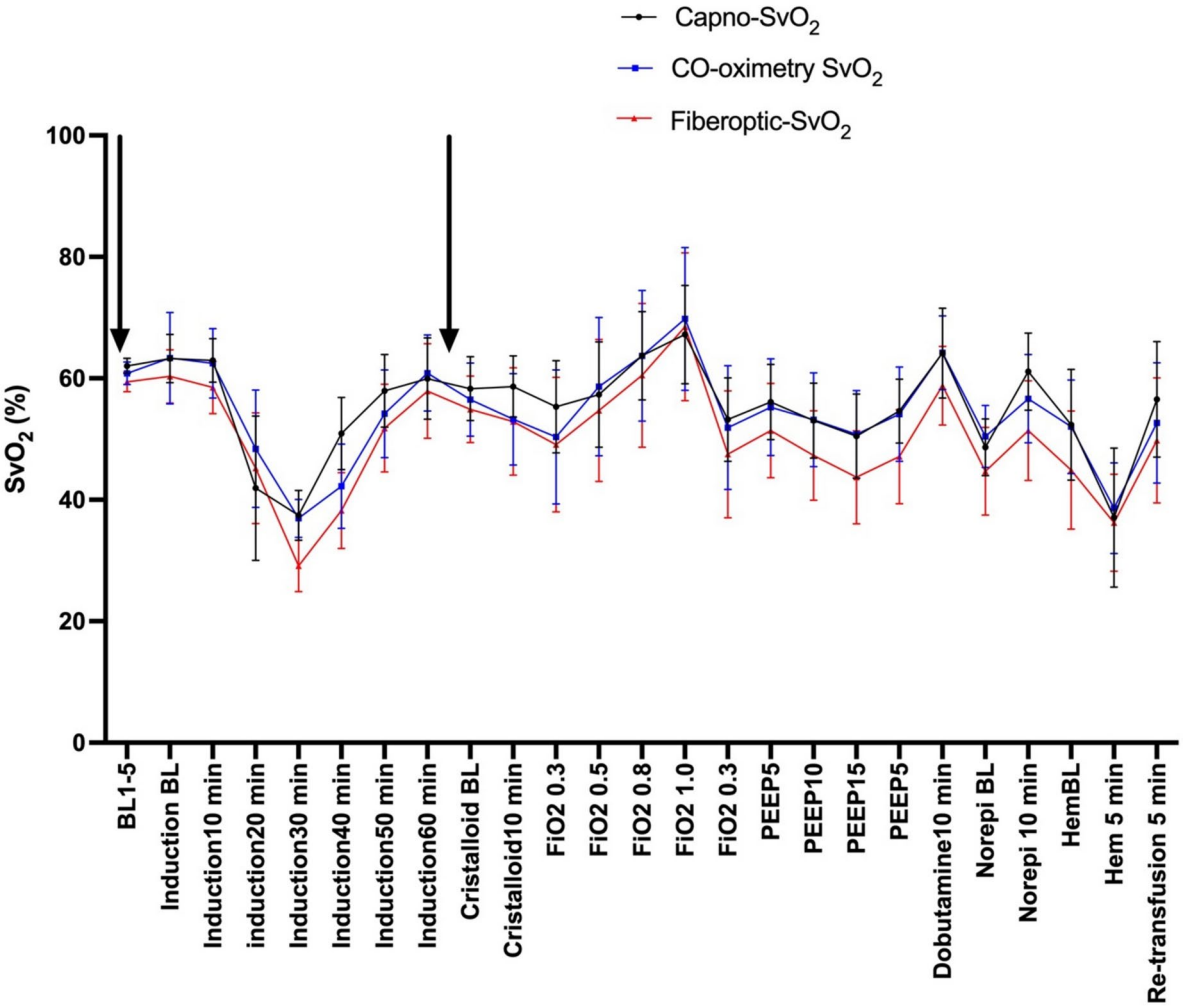
Event plot showing SvO_2_ responses to the various hemodynamic challenges for all three SvO_2_ monitoring methods. Values are mean (95% CI). N = 10 (refers to number of animals). Vertical black arrows indicate recalibration points for fibreoptic SvO_2_. BL1-5: Precision Baseline, Induction BL: Baseline before induction sepsis, FiO_2_: Fraction of inspired oxygen; PEEP: Positive End Expiratory Pressure (cmH2O), Norepi; Norepinephrine, HemBL; Baseline before start hemorrhage.

The majority of the animals showed a profound hemodynamic response to LPS-infusion and 6 out of 10 animals needed temporary stabilizing actions as per the goal directed protocol (e.g., norepinephrine, increased PEEP, FiO_2_ and crystalloid fluid resuscitation). The animals typically stabilized hemodynamically after the initial hour after starting LPS, but a few needed interventions later in the protocol during PEEP and hemorrhage interventions. The crystalloid fluid bolus resulted in a slight decrease in SvO_2_, as measured by the reference method, while the rest of study protocol following the endotoxemic induction affected SvO_2_, as measured by all methods, in a physiologically anticipated manner. The changes in SvO_2_ for each hemodynamic intervention is depicted in the event plot (Fig. 1).

### Agreement of absolute values

**Fig. 2:**
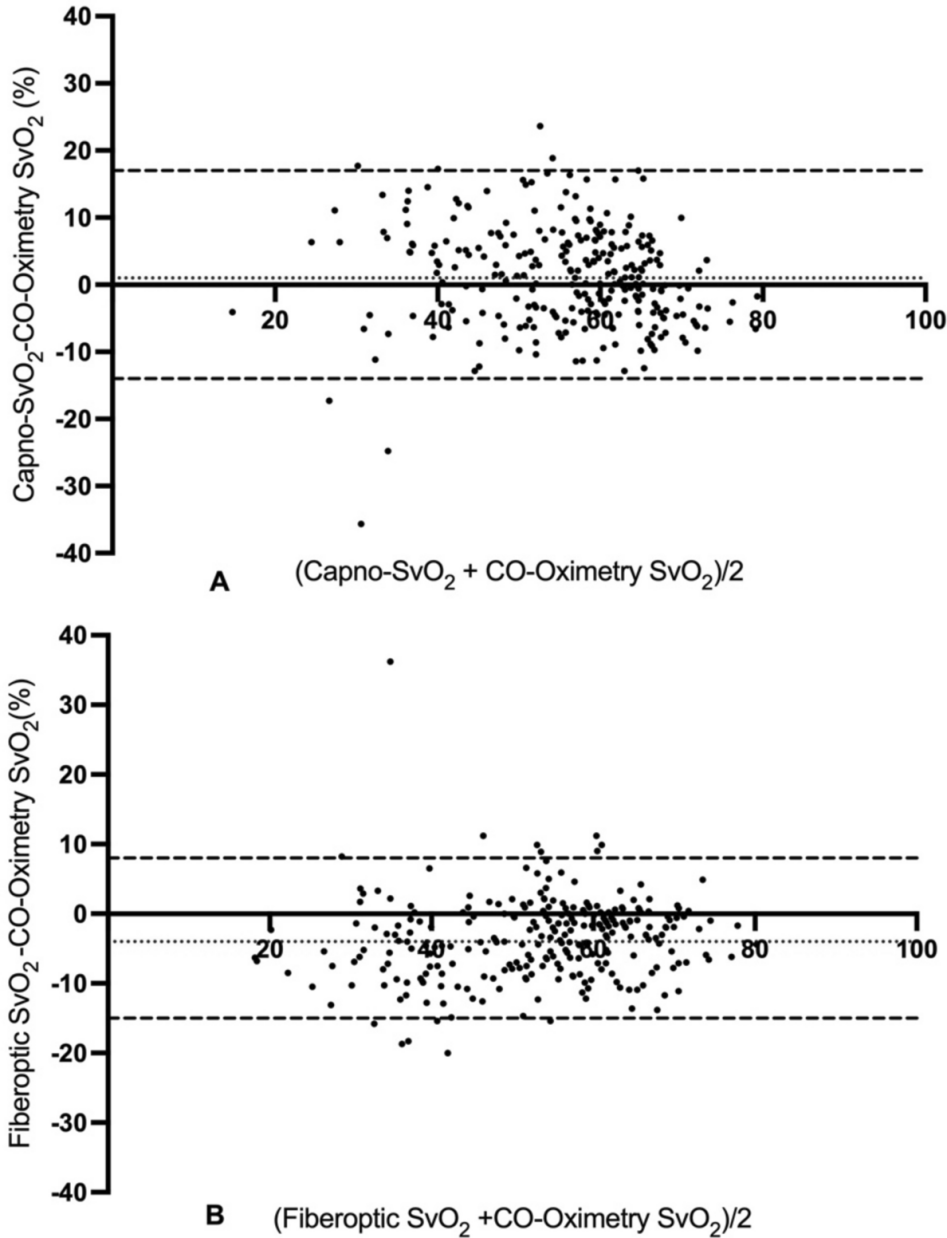
Bland–Altman plots for pooled recordings for Capno-SvO_2_ vs. CO-oximetry (**A**) and fiberoptic SvO_2_ vs. CO-oximetry (**B**). Dotted black line represents bias and black broken lines represent upper and lower limits of agreement. N = 10. A: 275 paired data points, B: 275 paired data points.

A Bland–Altman analysis was performed for all paired data points and showed a bias between Capno-SvO_2_ and Co-oximetry SvO_2_ of + 1% with 95% limits of agreement were -14% (95%CI-22 to -10%) to + 17% (95%CI 13 to 25%). The corresponding bias between fiberoptic SvO_2_ and CO-oximetry SvO_2_ was -4% with 95% limits of agreement -15% (95%CI -17 to -14) to + 8% (95%CI 7 to 10%). All values refer to absolute percentage points. Bland–Altman plots for both Capno-SvO_2_ and fiberoptic SvO_2_ vs Co-oximetry SvO_2_ are displayed in Fig. 2. The agreement of absolute values for the sepsis induction and each hemodynamic intervention are described in Table 1.

**Table 1:** Agreement of absolute values for both Capno-SvO_2_ and fiberoptic SvO_2_ as compared to the reference method, for the various different phases of the experiment.

Hemodynamic intervention	Compared methods	Bias (percentage points)	ULOA (CI) (percentage points)	LLOA (CI) (percentage points)
Baseline/precision	Capno vs CO-oxi	1	12	– 10
	Fiber vs CO-oxi	– 2	7	– 11
Sepsis induction	Capno vs CO-oxi	1	19	– 18
	Fiber vs CO-oxi	– 4	6	– 14
Crystalloid bolus	Capno vs CO-oxi	4	15	– 8
	Fiber vs CO-oxi	– 1	11	– 13
FiO_2_	Capno vs CO-oxi	1	16	– 15
	Fiber vs CO-oxi	– 3	6	– 12
PEEP	Capno vs CO-oxi	1	15	– 14
	Fiber vs CO-oxi	– 6	5	– 17
Dobutamine	Capno vs CO-oxi	5	22	– 13
	Fiber vs CO-oxi	– 1	17	– 18
Norepinephrine	Capno vs CO-oxi	1	16	– 14
	Fiber vs CO-oxi	– 5	6	– 17
Hemorrhage	Capno vs CO-oxi	– 1	16	– 17
	Fiber vs CO-oxi	– 5	18	– 27
Restitution of whole blood	Capno vs CO-oxi	4	21	– 13
	Fiber vs CO-oxi	– 3	6	– 11

### Trending ability

**Fig. 3:**
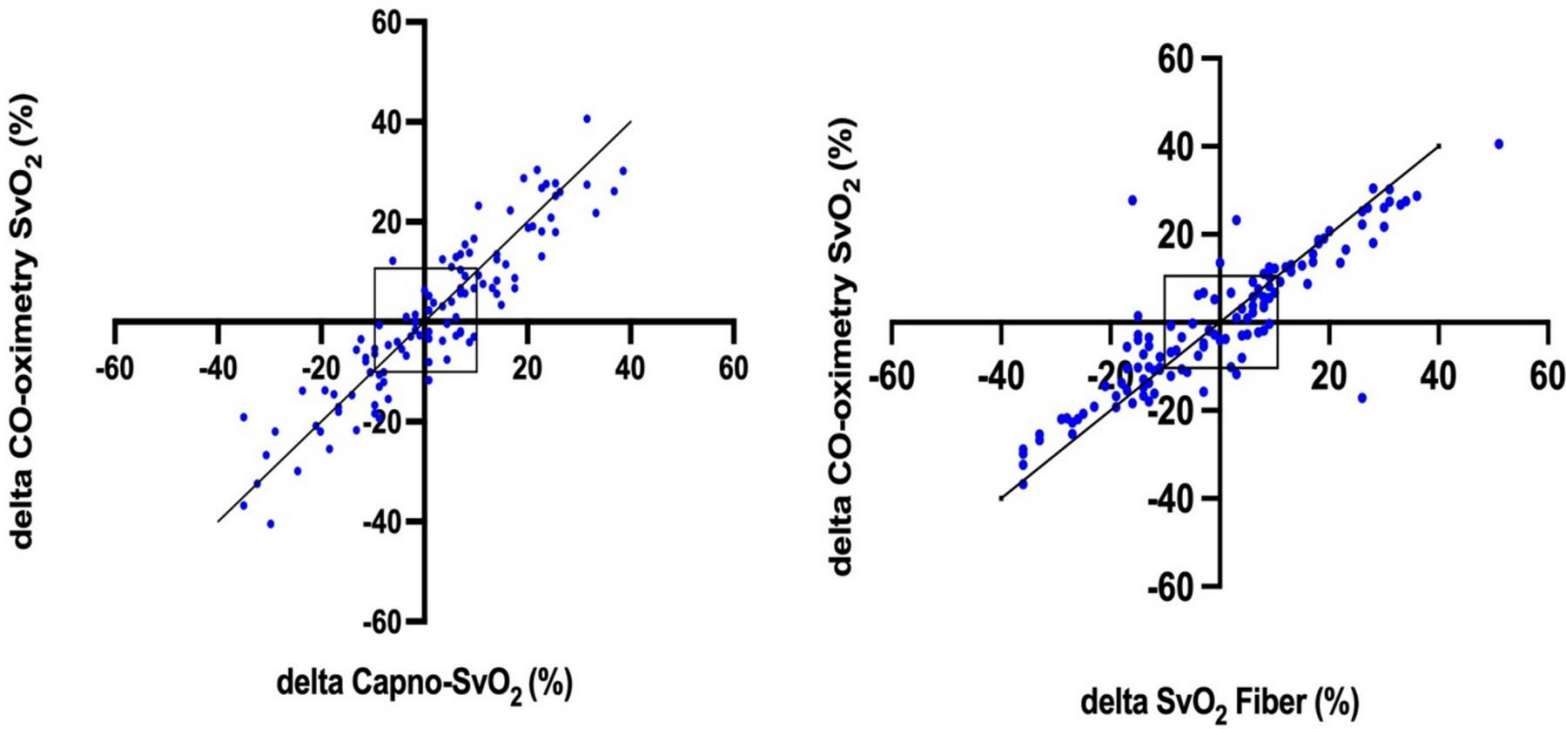
Four quadrant plots presenting the concordance between Capno-SvO_2_ and CO-oximetry SvO_2_ (**A**) and between fiberoptic SvO_2_ and CO-oximetry SvO_2_ (**B**). A: 70 paired delta values, B: 70 paired delta values. The central boxed area illustrates the 10% exclusion zone. Dotted line is line of identity. N = 10 (refers to number of animals).

The trending ability for Capno-SvO_2_ and fiberoptic SvO_2_ are presented in a 4-quadrant plot, Fig. 3. For Capno-SvO_2_, 70 datapoints were found outside the exclusion zone. Of these, 68 changed in the same direction as the reference method producing a concordance rate of 97% (95%CI 95 to 99%). Corresponding figures for fiberoptic SvO_2_ were a total of 70 where 5 were moving in the opposite direction, resulting in a concordance rate of 93% (95%CI 88 to 96%).

## Discussion

The main finding of the current study was that Capno-SvO_2_ generates average absolute values close to the gold standard CO-oximetry with relatively wide limits of agreements, although still near the predefined limit of 15 percentage points. In addition, Capno-SvO_2_ has a concordance rate of 97% against CO-oximetry and demonstrates a slightly better ability to detect change than the invasive fiberoptic SvO_2_with 93%,

### Agreement of absolute values between CO-oximetry and the tested methods

The tested methods showed a low variance during stable baseline conditions, with lower calculated inherent precision than the reference method. For the co-oximetry however, the inherent precision was somewhat higher than previously found using a similar model^1^. The Bland–Altman analysis showed a low mean difference between CO-oximetry and Capno-SvO_2_ (bias 1%), whereas the fiberoptic module slightly underestimated the SvO_2_ on average (bias -4%). The 95% limits of agreement for Capno-SvO_2_ were higher than previously found during major hemodynamic changes during non-sepsis conditions but were still close to the preset acceptable level of agreement of a maximum of 15%. In a similar manner, fiberoptic SvO_2_ also displayed a wide spread of the limits of agreement but still with values within the preset acceptable limit.

During stable baseline conditions the agreement between Capno-SvO_2_ and the reference method was within acceptable limits. However, this relation was negatively affected by the introduction of toxin, where bias and limits of agreements increased as outlined in Table 1. Possible reasons are discussed further below. The disturbances were maintained throughout the treatment protocol even though certain maneuvers were associated with better agreement.

Several potential reasons for the wider spread of data points than previously reported with similar protocols using non-sepsis hemodynamic scenarios can be identified.

Firstly, even if the Bland–Altman analysis is considered a standard statistical tool when comparing different monitoring systems, it may not be ideal for the assessment of rapid physiological changes^29^. Ideally, a study protocol would allow for the biological system to reach equilibrium after hemodynamic interventions and, subsequently, make recordings once stable conditions are reached^29^. A problem arises when studying unstable subjects with different monitoring techniques characterized by different response times. Theoretically, a new monitoring device producing the exact same values as the reference method, but with a time delay, would therefore in the presence of severe physiological instability, perform poorly in a Bland–Altman plot (explicatory figure available in supplements).

**Fig. 4:**
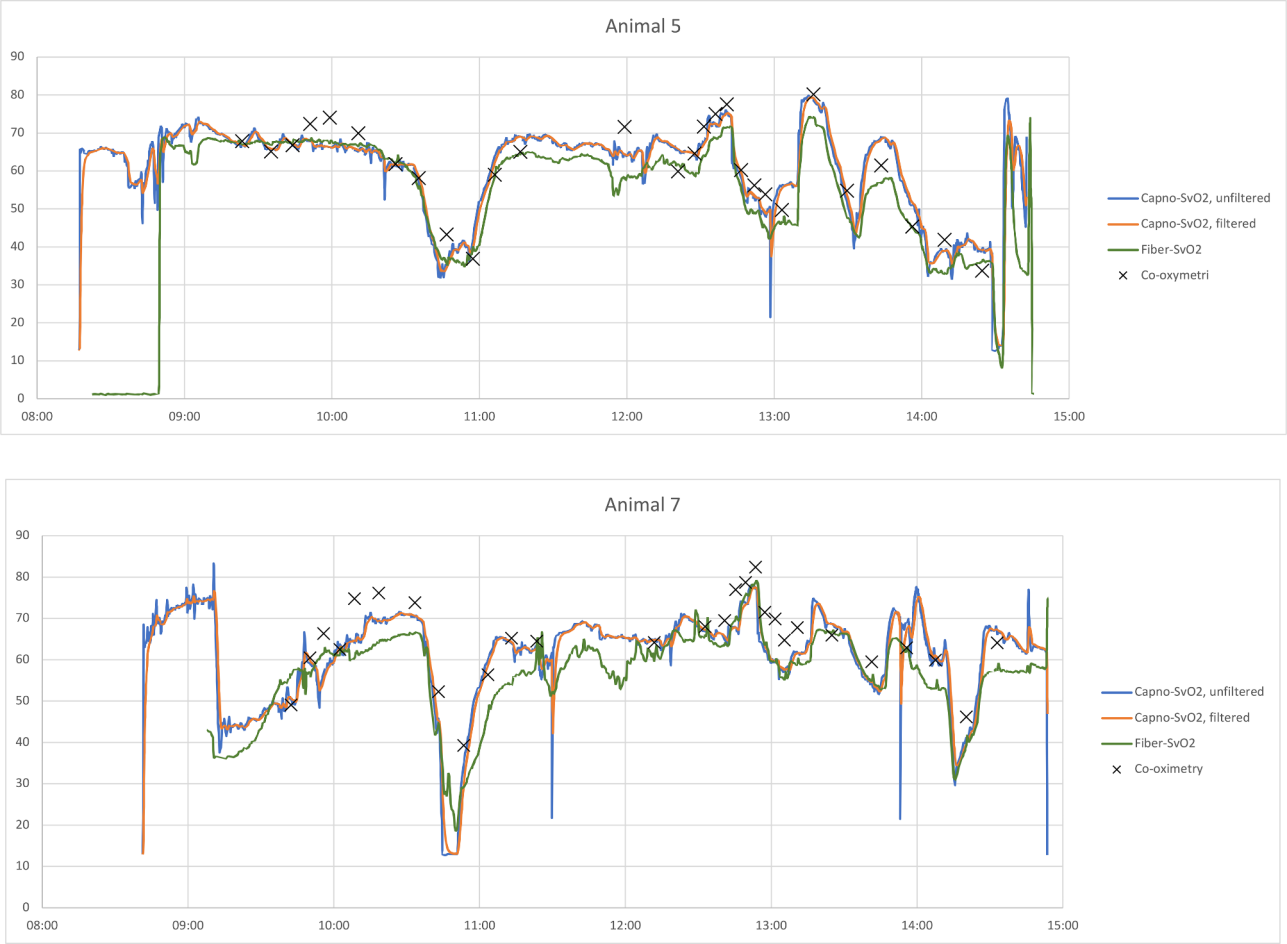
Continuous data from animal nr 5 and 7. Blue line represents unfiltered Capno-SvO2 (updated breath by breath), orange line represents Capno-SvO2 filtered as a moving mean over 50 s, green line represents Fiberoptic SvO2 and black crosses represents blood gases from the PAC.

When comparing Capno-SvO_2_ to fiberoptic SvO_2_ and CO-oximetry from a pulmonary artery catheter, it is therefore important to emphasize the different nature of these methods. Whereas the two latter are based on measurements from a catheter positioned in the pulmonary artery, Capno-SvO_2_ is a mathematically produced number based on continuous measurement of EPBF and FiO_2_ as described in the methods section. This explains some special features of the Capnodynamic method. In the present capnodynamic algorithm, EPBF is calculated as a moving mean of the nine last breaths and continuously updated for each new breath. Capno-SvO_2_, in turn, is shown in real time based on the present values of EPBF and FiO_2_ and is displayed as if the biological system had already reached equilibrium. This means that sudden variations in EPBF and FiO_2_ will produce a change in Capno-SvO_2_ immediately, while the in vivo change of actual SvO_2_ would be reached after a certain time delay. To exemplify this, we made simultaneous SvO_2_ recordings of all three methods just before and five minutes after euthanasia in one animal; Capno-SvO_2_ decreased from 29 to 0% (due to the sudden interruption of recorded breath-by-breath VCO_2_ and therefrom deduced EPBF) within 30 s after cardiac arrest while fiberoptic and CO-oximeter SvO_2_ increased from 37 to 41% and fiberoptic SvO_2_ decreased from 44 to 38%. The same rationale applies to some data points recorded in hemodynamically unstable animals during certain maneuvers (i.e. endotoxemic induction, PEEP elevation and hemorrhage) where Capno-SvO_2_ briefly displayed very low numbers, sometimes even zero. If desired, it would be possible to present Capno-SvO_2_ as a moving mean of e.g. 50 s rather than nine breaths, to make the method slightly less reactive in situations with rapid hemodynamic change. Also, introducing a delay to compensate for the difference in response time between CO-oximetry and Capno-SvO_2_ is a possibility, even though this was not further investigated in this study. In Fig. 4, individual continuous data for Capno-SvO_2_ and fiberoptic SvO_2_, are presented for animals nr 5 and nr 7. Capno-SvO_2_ is shown both in the original version and as a filtered (moving mean over 50 s) version. CO-oximetry data are plotted for reference.

Looking at the agreement of absolute values for different parts of the study protocol (Table 1), the wide limits of agreement during the endotoxemic induction are noticeable. Based on the reasoning above, this is not unexpected since the recordings are done strictly every tenth minute during a phase where many animals were highly hemodynamically deranged. This, as opposed to making an intervention, waiting ten minutes for stabilization and then record the data.

### Ability to detect change

As in previous studies, Capno-SvO_2_ showed a reliable ability to detect change (concordance rate 97%) when compared to the reference method. This is reassuring since tracking changes is important in the clinical setting. Fiberoptic SvO_2_ displayed a concordance rate of 93% which is also above the limit of the preset target of clinical reliability (i.e., > 92%)^27^. The higher than anticipated inherent precision of the reference method resulted in a somewhat large exclusion zone (10%) used for the concordance analyses. An exclusion zone of 5% would in this material result in a concordance rate of 95% for Capno-SvO2. This could be of interest for the reader since also smaller changes in SvO_2_ can be of clinical importance.

## Limitations

Firstly, the current study uses an experimental model of endotoxemia associated with a relatively fast acceleration of hemodynamic deterioration. In the clinical context, severe sepsis is unlikely to lead to such profound reactions already within 20 min of contact with the infecting agent. In addition, the experimental sepsis model used produces a decrease in SvO_2_ and not an increase in SvO_2_ as can be seen with mitochondrial dysfunction^30^. It is therefore not possible from the current study to fully conclude how the capnodynamic method performs in a situation with high SvO_2_ resulting from mitochondrial dysfunction.

Secondly, as discussed above the response time of the tested method may interfere with its ability to generate absolute values timed with values obtained from CO-oximetry. This could potentially be compensated for using a time delay even if this was not investigated in detail in the current study. A similar discussion, regarding the difference in response time between a test method and reference method, has previously been debated in the context of cardiac output monitoring^29^.

Thirdly, the goal-directed therapy protocol used to ensure the survival of the subjects may have led to differences in hemodynamic status between animals. This is likely due to an individual response to LPS, generating variations in inflammatory and hemodynamic response, mirroring the characteristics of sepsis in the clinical setting where the reaction can differ substantially between individuals. However, we believe that the main aim of the study, i.e., comparison of absolute values can still be viewed as valid.

Lastly, in this study RQ was assumed stable throughout the experiment, which might not be true for subjects developing sepsis or under treatment in the intensive care unit. Since reliable RQ measurement is cumbersome to perform and not readily available in most clinical settings we chose to study the performance of Capno-SvO_2_ using a single, preset RQ value. Ideally, RQ would have been individually measured multiple times throughout the experiment and future monitoring techniques making this feasible could possibly improve the accuracy of Capno-SvO_2_. Nevertheless, the mostly low variation of bias of Capno-SvO_2_ between different phases of the experiment (Table 1), indicates a minor influence of shifting RQ under these study settings. A higher or lower RQ value would shift the calculated SvO_2_, in the same direction but would not affect the spread of datapoints, i.e., the limits of agreement.

### Clinical applicability

Despite that Capno-SvO_2_ in its current form may not fully replace the use of a pulmonary artery catheter for SvO_2_ monitoring, the method could potentially play a role where invasive assessment of SvO_2_ is not feasible or warranted. This could be due to patient characteristics (i.e. pediatric patients), availability, staffing, or training issues. Furthermore, initiating Capno-SvO2 monitoring on an already intubated patient under controlled ventilation conditions is fast and normally uncomplicated.

It is also important to emphasize that the preset acceptable limits of agreement of + /- 15 percentage points do not necessarily apply in clinical context where such divergence might be unacceptable. The wider limits of agreements for Capno-SvO_2_ in this sepsis model, as compared to previous published data, is probably at least partly due to differences in response time in the investigated monitoring techniques, as lined out in the previous text. However, this was not á priori addressed in the experimental setup and individual, absolute values of Capno-SvO_2_ should be interpreted cautiously and in conjunction with other available hemodynamic parameters for the subject. On the other hand, Capno-SvO_2_ reliably detects changes and has the potential to early identify significant hemodynamic change, also in subjects with sepsis.

In its current form, Capno-SvO_2_ requires an intubated patient and controlled ventilation in order to generate controlled fluctuations in exhaled CO2, which may be a limitation for widespread use. However, these prerequisites would often already be met when a subject is deemed in need of SvO_2_ monitoring.

## Conclusions

In the current experimental endotoxemic model, continuous, non-invasive Capno-SvO_2_ generates average absolute values close to the gold standard CO-oximetry, but with relatively wide limits of agreement. Furthermore, Capno-SvO_2_ displayed a concordance rate of 97% compared to CO-oximetry and exhibits slightly better performance in trending ability compared to invasive fiberoptic SvO_2_.

## Electronic supplementary material

Below is the link to the electronic supplementary material.

Supplementary Information 1.

Supplementary Information 2.

Supplementary Information 3.

## Data Availability

Data will be available from the corresponding author upon reasonable request.

## Abbreviations

SvO_2_: Mixed venous oxygen saturation
PAC: Pulmonary artery catheter
VO_2_: Oxygen consumption
FiO_2_: Fraction of inspired oxygen
VCO_2_: CO_2_ elimination rate
LPS: Lipo-polysaccharide
PEEP: Positive end expiratory pressure
EPBF: Effective pulmonary blood flow
RQ: Respiratory quotient
CcO_2_: Pulmonary end capillary oxygen content
CvO_2_: Oxygen content in mixed venous blood
