# Supplementary material for: Capnodynamic assessment of mixed venous oxygen saturation in a porcine experimental endotoxemic model
Source: Sci Rep. 2024 Nov 5;14:26807. doi: 10.1038/s41598-024-77483-7 (PMC11538446; doi:10.1038/s41598-024-77483-7)
Supplement: Supplementary file 1 — Supplementary Information 1. [file 41598_2024_77483_MOESM1_ESM.pdf]

Below an example of CO2 fraction [%] and flow [lpm] versus time [min] from a sequence of breaths is shown.

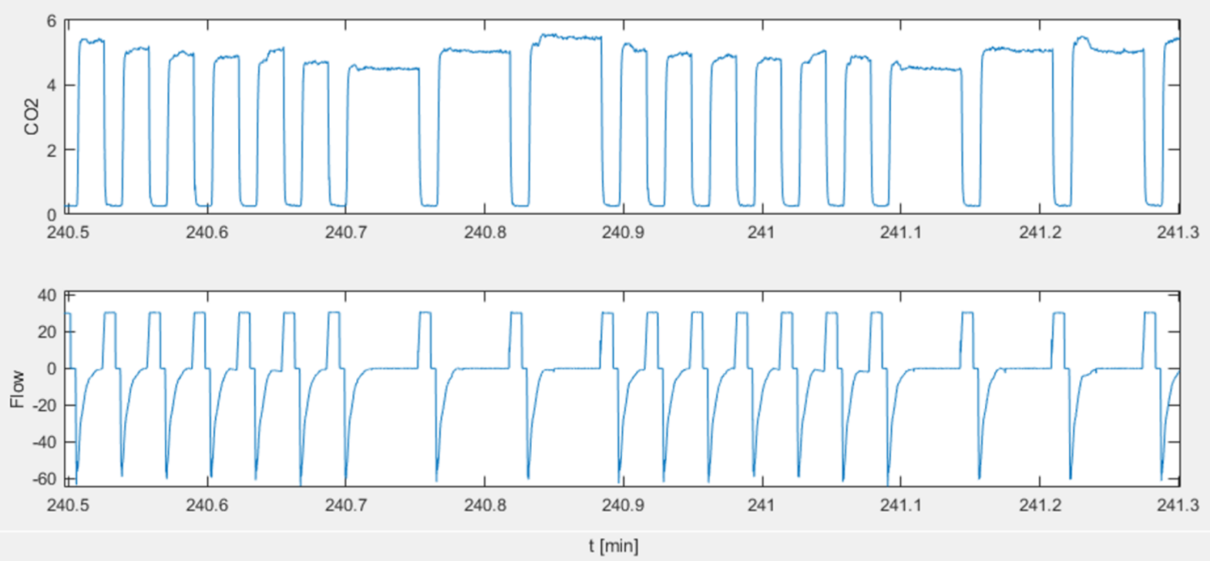

CO2 and flow are combined to create volumetric capnograms. The figures below show nine such capnograms over a sequence of nine breaths in alternating ventilation cycle, and how the alveolar CO2 level varies up and down (blue dots), and with the fitted model adapted to that variation (red line segments).

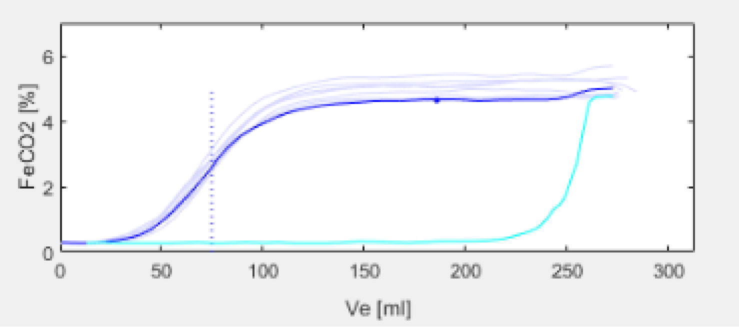

Volumetric capnogram. The cyan blue curve is the inspiratory branch.

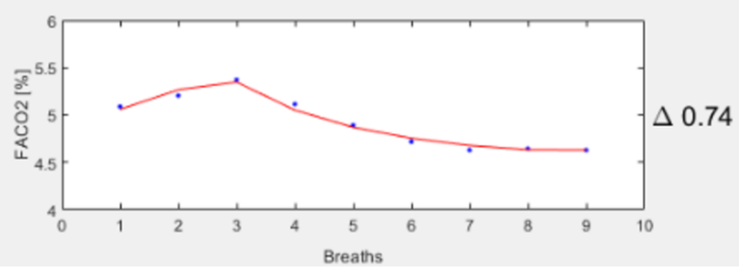

Alveolar variation over nine breaths (blue dots) and capnodynamic adaption (red line).

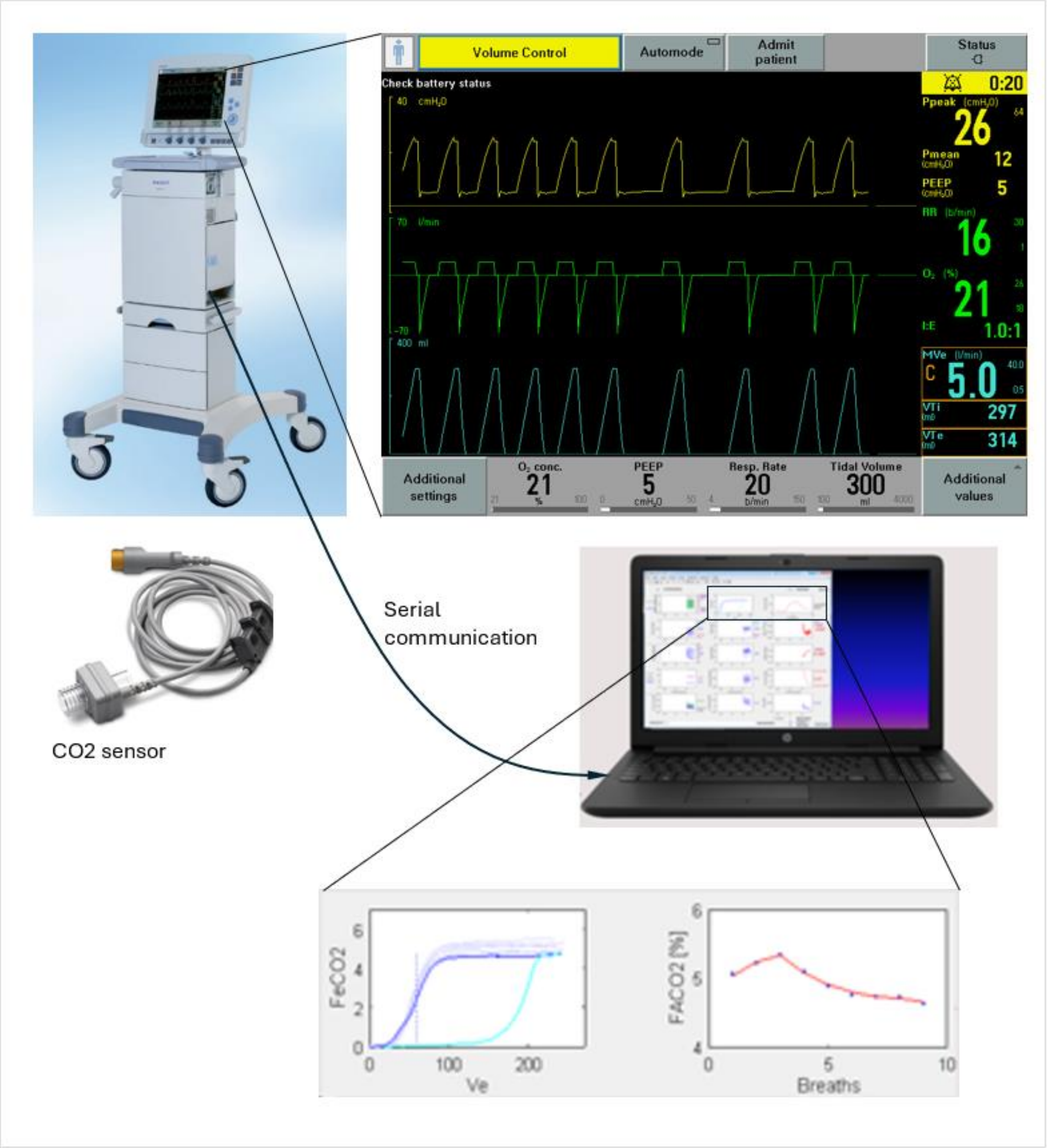

The prototype system. Servo-i with varying ventilation, mainstream CO2 sensor, laptop connected via serial communication with software to analyse volumetric capnography and capnodynamic equations.
