# Supplementary figures and images for: Capnodynamic assessment of mixed venous oxygen saturation in a porcine experimental endotoxemic model
Source: Sci Rep. 2024 Nov 5;14:26807. doi: 10.1038/s41598-024-77483-7 (PMC11538446; doi:10.1038/s41598-024-77483-7)

# Effect of different response time

Paired recording

SvO2 B  
62.5

SvO2 A  
48

SvO2

SvO2 A  
SvO2 B

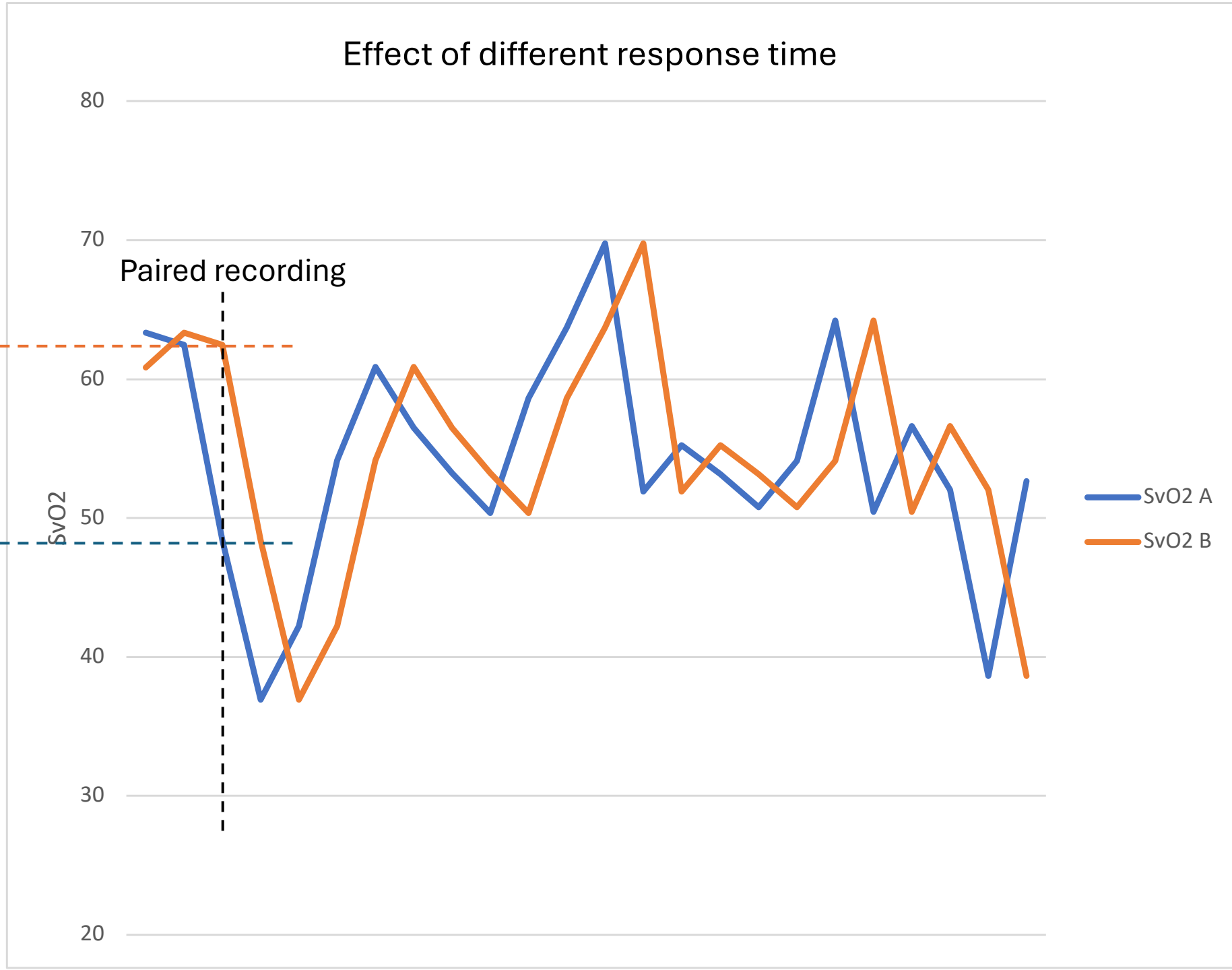

Supplement: Supplementary file 2 — Supplementary Information 2. [file 41598_2024_77483_MOESM2_ESM.pdf]
