# Supplementary material for: Capnodynamic assessment of mixed venous oxygen saturation in a porcine experimental endotoxemic model
Source: Sci Rep. 2024 Nov 5;14:26807. doi: 10.1038/s41598-024-77483-7 (PMC11538446; doi:10.1038/s41598-024-77483-7)
Supplement: Supplementary file 3 — Supplementary Information 3. [file 41598_2024_77483_MOESM3_ESM.pdf]

Parameter	Threshold value for intervention	Interventions
PaO_2	<10 kPa first time	Increase FiO_2 to 0.45
PaO_2	<10 kPa thereafter	1. Increase FiO_2 to the next level: 0.6→0.8 →1.0, and<br>2. Increase PEEP to the next level: 5→8→10→14 cmH_2O, and<br>3. Lung recruitment maneuver[a]
PaO_2	>20 kPa	Decrease FiO_2 to the previous level: 1.0→0.8→0.6→0.45→0.3
$PaCO_2$	>6.5 mmHg	Increase V_T with 10 %, to maximum 15 mL x kg^{-1}
$PaCO_2$	<4.5 mmHg	If respiratory rate is ≤25, decrease V_T with 10 %. If respiratory rate is >25, decrease it with 10 %.
MAP (<60 min)	MAP=MPAP (<60 min after start of *LPS*-infusion)	Single dose of 40 µg norepinephrine i.v.
	MAP<50 mmHg (<60 min after start of *LPS*-infusion)	Start norepinephrine infusion (20 µg x mL^{-1}) 5 mL x h^{-1}. If ongoing norepinephrine infusion, increase rate one step[b].
MAP (>60 min)	MAP<60 mmHg (>60 min after start of *LPS* infusion)	Start norepinephrine infusion (20 µg x mL^{-1}) 5 mL x h^{-1}. If ongoing

		norepinephrine infusion, increase rate one step[b].
CO	CO<2.0 L x min^{-1}	Fluid bolus with acetated Ringer's solution 15 mL x kg^{-1} and/or start norepinephrine infusion (20 µg x mL^{-1}) 5 mL x h^{-1}. If ongoing norepinephrine infusion, increase rate one step[b].
MAP/MPAP	MAP = or < MPAP (>60 min after start of *LPS*-infusion)	1. Single dose of 20 µg norepinephrine i.v., and<br>2. Start norepinephrine infusion (20 µg x mL^{-1}) 5 mL x h$^{-1,}$ or, if ongoing norepinephrine infusion, double the infusion rate, (5→10→20→40 mL x h^{-1})<br>3. Fluid bolus with acetated Ringer's solution 15 mL x kg^{-1}
MAP	MAP>100 mmHg	If ongoing norepinephrine infusion (20 µg x mL^{-1}), decrease rate one step: 40→20→10→5→0 mL x h^{-1}
Blood Glucose	<4.0 mmol x L^{-1}	Give 20 mL of 30 % glucose-solution i.v.

[a] PEEP was increased stepwise until a peak pressure of 35 cm H_2O was reached. Then, an inspiratory hold was performed for 10 s. Thereafter, the PEEP was stepwise decreased to the PEEP defined by the protocol. If MAP decreased to the level of the MPAP, the recruitment manoeuvre was aborted.
